# Expanding educational opportunities virtually in the time of COVID-19

**DOI:** 10.5116/ijme.6096.a0db

**Published:** 2021-05-27

**Authors:** Madelon L. Finkel, Silke Anna T. Weber, Karina Luiz Chamma

**Affiliations:** 1 Department of Population Health Sciences, Office of Global Health Education, Weill Cornell Medicine, New York, NY, USA; 2 Department of Ophthalmology, Otolaryngology and Head and Neck Surgery, International Office of Botucatu Medical School, State University São Paulo, UNESP, Botucatu Sao Paulo, Brazil; 3 International Office of Botucatu Medical School, State São Paulo, UNESP, Botucatu Sao Paulo, Brazil

**Keywords:** Educational opportunities, virtual platform, COVID-19, USA

## To the Editor

As the rapid spread of the coronavirus continues to rage around the world, most school systems have suspended in-class instruction in an effort to stem the spread of the virus and to ensure the health of teachers and learners. Secondary, undergraduate, and graduate schools had to quickly pivot to a mix of blended learning (mix of online and in-class instruction). While blended learning is not a new concept per se, by necessity, it is becoming the ‘new normal’ one year into the pandemic. In particular, COVID-19 is causing an unprecedented disruption in medical education.

For over the past decade, medical schools have been exploring a number of ways to improve the teaching of core basic and clinical sciences, including eliminating/reducing lectures; using technology to replace/enhance anatomy and laboratories; implementing team-facilitated, active, and self-directed learning; and decreasing the basic science curriculum to 12 or 18 months.^1^  However, the 2020-2021 COVID-19 pandemic profoundly altered how educators teach, forcing the rethinking of how students should be taught.^2^ While most medical schools already had some online component in their curriculum, the pandemic necessitated a quick transformation from in-class learning to online learning.

Overwhelmingly, medical schools pivoted from in-class instruction to an e-learning format in the Spring 2020 semester, with many suspending clinical rotations. Online teaching by Zoom or other similar online platforms became the norm. Lectures and small-group learning activities were presented virtually, while clinical skills sessions either moved online or relied on telemedicine.^3^  While each medical school had to figure out how to redesign its curriculum, in the United States, for example, the American Medical Association (AMA) compiled and made available virtual and remote learning resources to help medical educators decide on the best ways to teach remote learners during the coronavirus pandemic.^4^  The American Association of Medical Colleges (AAMC) also made available guidance and resources, including guidance on medical students’ participation in the clinical setting during the COVID-19 pandemic, transition to residency, and early graduation for fourth-year medical students.^5^

While the transition to virtual learning using online platforms for the teaching of basic science and clinical medicine was adopted by medical schools fairly successfully, some programs and electives were shut down. Specifically, most medical schools have a global health program, which combines educational offerings and clinical experience abroad. International clinical electives programs were suspended due to the coronavirus travel bans imposed by individual medical schools as well as countries around the world. International students could not come to the United States to take clinical electives, and US students could not travel abroad.

While it is difficult to maintain a global health program in the age of COVID-19, it is not impossible, and new, creative ways of continuing the program emerged during the lockdown. Challenges can serve to stimulate thinking in ways that previously may not have envisioned.^6^^,^^7^^,^^8^ The following describes how Weill Cornell Medicine and its partners in Latin America took the opportunity to expand its global learning educational program during COVID-19.

The Weill Cornell Medicine’s Office of Global Health Education (OGHE) oversees a robust global health program that includes didactic and applied experience opportunities to enable our medical students to better understand the complexities of cultural competence that are important and integral components of the medical school curriculum. OGHE oversees and coordinates the global health educational electives program for Weill Cornell medical students, as well as for visiting international medical students who wish to participate in a clinical elective at Weill Cornell Medicine. (For a more in-depth overview of the global health program, see Norris et al.)^9^

OGHE’s educational programs pre-COVID-19 were in-person events, including a ten-week Intro to Global Health seminar series, global health career seminar series, and monthly Global Health Grand Rounds. With in-person events cancelled during COVID-19 lockdown, OGHE quickly pivoted to an online virtual format to host its seminar series. OGHE also introduced a new virtual ten-week seminar series on global climate change. Lectures on a wide variety of issues were presented (e.g., the impact of global climate change on health, water resources, food supply, migration, air quality, vector-borne diseases, extreme weather).

By switching to a real-time, virtual format, we were able to offer our educational programs to a wider audience, including our global partners that served to expand our global educational reach. For those in different time zones, recorded sessions (password protected) could be accessed at any convenient time.

Pre-COVID-19, we partnered with the Faculty of Medicine UNESP Botucatu (Brazil) to present a live 4-week elective on evidence-based medicine and clinical decision-making to learners at Botucatu. This elective was co-taught by faculty at both institutions with in-person lectures. COVID-19 forced us to present the elective entirely via Zoom, which actually served to increase the number of individuals participating in each session. Learners who could not physically attend now could log on unrestricted by place. Realizing that our electives could be expanded to other interested parties, we invited our partner medical school in Lima, Peru, the Faculty of Medicine of the National University of San Marcos (SMSM), to participate in the clinical decision-making elective with Botucatu and Weill Cornell. This opportunity to include medical students and faculty in Lima dovetails with SMSM’s decision to expand its online teaching. Rather than create new electives, the leadership at SMSM decided to join existing electives. Instruction is in English, and small group workgroups are composed of Brazilian, Peruvian, and American medical students. 

The sharing of educational curricula was viewed by our partners and us as a wonderful opportunity to connect using a virtual platform. By doing so, we collectively not only expanded our academic offerings but also enabled learners from different countries to learn together in real-time. While the impetus for this experiment was COVID-19, there is no doubt that once the pandemic passes, we will continue to offer these, and perhaps newly created seminars, in real-time to our global partners.

### Conflict of Interest

The authors declare that they have no conflict of interest.
